# Histopathological Analysis of Pseudoexfoliation Material in Ocular Surgeries: Clinical Implications

**DOI:** 10.3390/diagnostics14192187

**Published:** 2024-09-30

**Authors:** Laura Denisa Stejar, Anca-Maria Istrate-Ofițeru, Ioana Teodora Tofolean, Dana Preoteasa, Florian Baltă

**Affiliations:** 1 Department of Ophthalmology, Carol Davila University of Medicine and Pharmacy, 050474 Bucharest, Romania; 2 Onioptic Hospital of Ophthalmology, 200136 Craiova, Romania; 3 Department of Ophthalmology, Clinical Emergency Eye Hospital, 010464 Bucharest, Romania; 4 Research Centre for Microscopic Morphology and Immunology, University of Medicine and Pharmacy of Craiova, 200349 Craiova, Romania

**Keywords:** histopathology, pseudoexfoliation syndrome, anterior lens capsule, eyelid, conjunctiva

## Abstract

Background: Pseudoexfoliation syndrome (PEX) is a common age-related ocular condition characterized by the accumulation of a fibrillar, pseudoexfoliative material on the anterior segment of the eye. This study aims to investigate the histopathological characteristics of pseudoexfoliative material within different ocular structures, including the eyelid, conjunctiva, and anterior lens capsule. Methods: A total of 32 anterior lens capsules, 3 eyelid fragments, and 12 conjunctival specimens were obtained from patients clinically diagnosed with PEX during ocular surgeries at the Onioptic Hospital of Ophthalmology. The tissue specimens were subsequently processed using the classical histological technique of paraffin embedding. This process enabled the production of serial sections with a thickness of 4 microns, facilitating the microscopic examination of fine details. The sections were stained with the hematoxylin-eosin (HE) method for the observation of microscopic structures. Results: This study’s findings reveal that PEX material, characterized by its fibrillar and amorphous components, is consistently present across multiple ocular structures, including the anterior lens capsule, eyelid, and conjunctiva. When stained with H&E, the PEX material typically appears as amorphous, eosinophilic deposits. Under higher magnification, these deposits exhibit a fibrillar structure, often appearing as irregular, granular, or filamentous aggregates. Conclusions: The deposit of fibrillar material in the eyelid and conjunctiva, though less commonly emphasized compared to other structures, is a significant finding that sheds light on the systemic nature of the syndrome. The consistent identification of fibrillar eosinophilic deposits across these structures highlights the systemic distribution of PEX material, reinforcing the notion that PEX syndrome is not confined to the anterior segment of the eye.

## 1. Introduction

Pseudoexfoliation syndrome (PEX) is a significant age-related condition characterized by the abnormal production and deposition of fibrillar extracellular material on the anterior segment of the eye. This syndrome encompasses a spectrum of ocular, surgical, and systemic complications, making it a multifactorial clinical entity. The clinical appearance of pseudoexfoliation syndrome dates back to 1917, when it was first characterized by John Gustaf Lindberg as deposits of granular material at the pupillary margin of the iris and on the anterior surface of the lens [1].

The global incidence varies considerably in reported studies, with prevalence documented as between 1.5% and 40.9% worldwide [2,3]. Demographic studies have demonstrated a predisposition in certain geographic areas and ethnic groups [4]. The prevalence of PEX ranges from 3.6% to 34.2% in European countries, from 1.5% to 22.1% in Asian countries, and from 1.5% to 40% in African countries, suggesting a general lack of consensus regarding these epidemiological studies [2,3,5,6,7].

Pseudoexfoliation material (PXM) is particularly concerning in the context of ocular surgeries, such as cataract extraction, due to its association with numerous complications, including zonular weakness, increased intraocular pressure, and challenges during surgery [8,9]. One meta-analysis based on 22 case-control, observational, and cohort studies concluded that there is a two-fold increased risk of intraoperative posterior capsule rupture or zonular dialysis in patients with pseudoexfoliation during cataract surgery performed by phacoemulsification [10]. Similarly, a statistically significant difference has been demonstrated regarding postoperative complications such as corneal edema, intraocular hypertension, and postoperative uveitis [11]. It also plays a role in the development of pseudoexfoliative glaucoma when accumulated in the trabecular meshwork [9]. Rao A et al. demonstrated a correlation between a pattern of lens deposits and increased intraocular pressure, indicating that PEX is a progressive disease in which PXM passes through multiple appearance stages [12]. Apart from anterior segment manifestation, studies have shown that PXM can also affect posterior segment structures such as macular vessel density and foveal avascular zones [13] and subfoveal choroidal thickness [14].

Systemic complications related to PXF syndrome include alterations in collagen and elastin within the vessel walls, leading to conditions such as hypertension, myocardial infarction, stroke, Alzheimer’s disease, and diabetes. Additionally, extraocular connective tissue disorders, including benign prostatic hyperplasia, chronic kidney disease, chronic obstructive pulmonary disease, and inner ear dysfunctions, have also been associated with PXF syndrome [2,15,16,17,18,19]. Although the literature is abundant regarding the increased risk of complications, there is a lack of data on the mechanisms of their occurrence and how they could be predicted.

Histopathological examination of PXM provides critical insights into its composition, distribution, and potential impacts on surgical outcomes. Various studies have emphasized the importance of analyzing this material to better understand its role in ocular pathologies and to develop targeted interventions [20,21]. The etiopathogenesis involves the formation of an exfoliative material that firmly adheres to the anterior lens capsule and the posterior epithelium of the iris and ciliary body, as well as the Zonule of Zinn and the anterior surface of the vitreous [20].

Detailed histological examinations have identified the presence of elastin-like fibers within PXM, which may contribute to the increased rigidity and fragility of the zonules, leading to complications during cataract surgery [22]. Additionally, immunohistochemical studies have demonstrated the presence of specific markers, such as amyloid P and clusterin, within PXM, which may be involved in the pathogenesis of secondary open-angle glaucoma, often associated with PEX [23,24].

Studies have shown that PXM consists of microfibrillar components, glycoproteins, and various types of collagen, all of which contribute to its adhesive properties and its capacity to induce structural alterations in ocular tissues [23]. Electron microscopy has revealed that PXM is not confined to the eye but is also present in other tissues, indicating the systemic nature of the disorder [25]. The exfoliative material is composed of characteristic fibrils with cross-banding, embedded in an amorphous matrix and found both within epithelial cells and associated with a disorganized, reduplicated basement membrane [26]. These findings suggest that the material originates from the epithelium of the lens, iris, and ciliary body, possibly as a result of an underlying metabolic disorder [26].

The precise nature and distribution of this material within the ocular structures are not fully understood, despite its significant clinical implications. It is believed to involve a protein core surrounded by glycoproteins, forming a proteoglycan/glycoprotein complex. Another important pathogenic mechanism includes the presence of ischemia, hypoxia, oxidative stress, and chronic inflammation [20,27,28]. It has not been clearly determined whether the accumulation of pseudoexfoliative material is due to excessive production or inefficient degradation [29]. There is evidence suggesting that PXM etiology is either excessive de novo synthesis [30] or improper degradation caused by an imbalance between matrix metalloproteinases and their tissue inhibitors [31,32,33].

This study aims to investigate the histopathological characteristics of pseudoexfoliative material within different ocular structures, including the eyelid, conjunctiva, and anterior lens capsule. By exploring the microscopic characteristics of PXM, we seek to elucidate the complexities of PEX and offer guidance for clinicians managing this challenging condition.

## 2. Materials and Methods

This is a prospective, interventional study carried out on 80 patients operated on at the Onioptic Hospital of Ophthalmology, Craiova, Romania in 2024 by the same surgeon. The study included patients older than 40 years who were clinically diagnosed with pseudoexfoliation syndrome and who underwent standard ophthalmic procedures. Patients under 40 years old, as well as those over 40 years old with increased surgical risk—such as a history of trauma, corneal opacities, Fuchs endothelial dystrophy, aphakia, previous vitreoretinal surgery, and other types of cataracts, or those unable to undergo standard procedures—were excluded. Written informed consent was obtained from all participants prior to the study.

**Figure 1:**
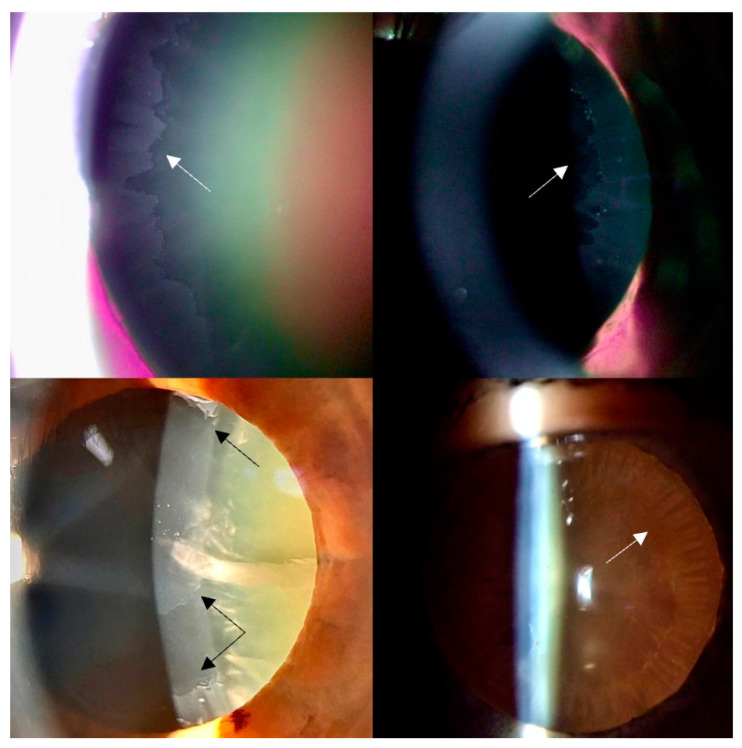
Slit-lamp biomicroscopy showing lens pseudoexfoliation associated with age-related cataracts. Arrows indicate pseudoexfoliative deposits on anterior lens capsule.

All patients were evaluated according to an ophthalmological protocol established prior to the commencement of the study. Demographic data (age, sex), medical history, and ophthalmological parameters were collected, including best-corrected visual acuity, intraocular pressure, corneal thickness, endothelial cell count, corneal curvature, anterior chamber depth, and biomicroscopic examinations of the anterior and posterior segment. Based on the slit-lamp examination, the diagnosis of pseudoexfoliation syndrome was confirmed if fibrillar material was observed on the anterior lens capsule, with a classic three-ring appearance, or on the pupillary ruff as white flaky “dandruff-like” deposits following pharmacologic mydriasis [1,8,34,35] (Figure 1).

Cataract diagnosis was established during biomicroscopic examination in accordance with the Emery–Little classification system for lens opacities [36]. Pterygium severity ranged from grade 1 to grade 4 depending on the extent of corneal involvement [37]. Ectropion [38] and entropion [39] grading scales were employed to establish surgical indications. Patients who met the surgical criteria and satisfied the inclusion criteria were enrolled in the study in successive order. Participants were required to provide informed consent for study participation.

For this histopathological study, anterior lens capsules were obtained during standard manual capsulorhexis in cataract surgery, eyelid fragments were provided during ectropion or entropion surgeries, and conjunctival specimens were collected during pterygium removal. Not all specimens were of sufficient quality to be examined. We analyzed 32 anterior lens capsules, three eyelid fragments, and 12 conjunctival specimens from patients clinically diagnosed with PEX during 47 ocular surgeries. The samples were preserved in 10 mL vials containing 5 mL of 10% neutral buffered formalin at a temperature between 15–25 °C until analysis. The subsequent analysis was carried out at the Research Center for Microscopic Morphology and Immunology Studies at the University of Medicine and Pharmacy of Craiova (UMFCV).

Following fixation in 10% formalin solution, the specimens were transferred to plastic histological cassettes and rinsed with tap water for 24 h to eliminate excess fixative from the tissues. Subsequently, they were embedded in purified histological paraffin, with a fixed melting point of 56 °C.

The paraffin embedding histological technique involves several key steps: dehydration of the tissues in increasing concentrations of alcohol—70%, 90%, 96%, and 100%—followed by clearing in xylene. The tissues are then infiltrated with purified paraffin and embedded and sectioned using the Microm HMB350 rotary microtome. Sections are affixed to histological slides, dried, and then stained. To ensure optimal tissue adherence, sections were applied to clean glass slides, as well as to slides treated with Poly-L-Lysine. The HMB350 microtome features a water-based section transfer system, which aids in achieving a uniform collection of serial sections with minimal loss. Staining of the tissue sections was performed using the hematoxylin-eosin (HE) method. Sections of 4 microns in thickness were prepared to facilitate the microscopic identification of fine histological details.

The statistical analysis was carried out using SPSS Statistics 26.0. Percentages, means, standard deviation (SD), and the 95% confidence interval (95% CI) were employed for the descriptive part of this study. Categorial nominal variables included biomicroscopic findings and continuous numeric variables were represented by age, pre-operative biometry data, and post-operative best-corrected visual acuity and intraocular pressure. The chi-square test was used to establish statistical significance at a cutoff value of *p* < 0.05.

The principles of the Declaration of Helsinki were followed in this study. Ethical approval was received beforehand from the Ethics Committee of Onioptic Ophthalmology Hospital (910/19 August 2024).

## 3. Results

The study included 80 eyes from 80 patients with an age range between 61 and 90 years old. The cohort consisted of 63.25% females and 36.75% males. The mean age was 78.57 ± 6.18 years for females and 76.70 ± 7.85 years for males.

Biometric results are illustrated in Table 1.

Pre-operative biomicroscopic exam characteristics are detailed in Table 2.

Best corrected visual acuity (logMar) was measured at presentation (baseline) and at the 7-day, 1-month, and 3-month follow-ups (Table 3).

**Figure 2:**
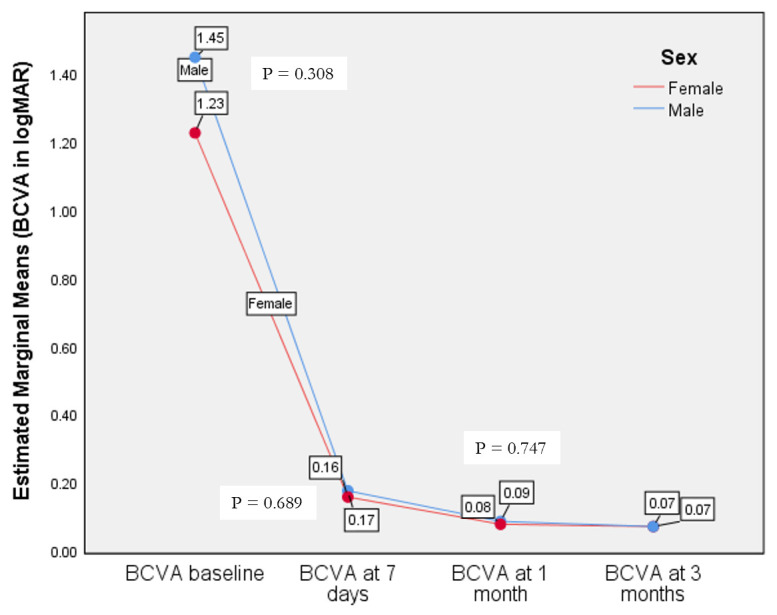
Best-corrected visual acuity in females versus males after cataract surgery in pseudoexfoliation patients.

There was no statistical difference between females and males in terms of BCVA outcomes (Figure 2).

Intraocular pressure (IOP) was measured at presentation (baseline) and at the 7-day, 1-month, and 3-month follow-ups (Table 4).

**Figure 3:**
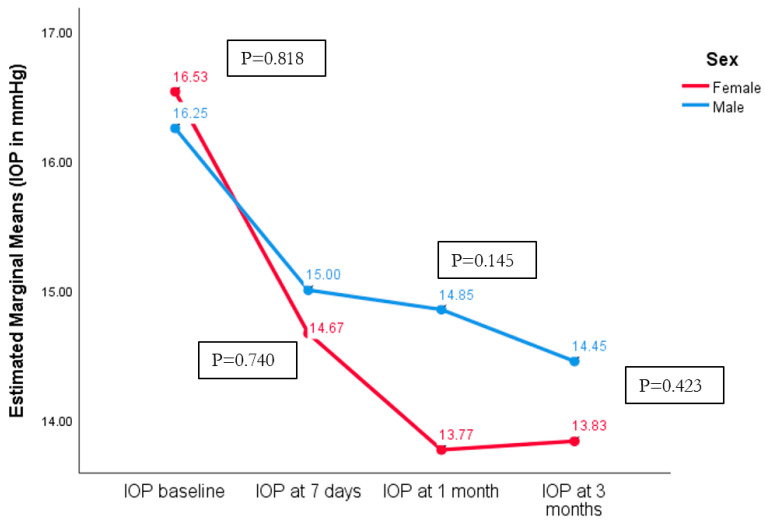
Intraocular pressure in females versus males after cataract surgery in pseudoexfoliation patients.

There was no statistical difference between females and males in terms of IOP outcomes (Figure 3).

The histopathological analysis of pseudoexfoliation material obtained from ocular surgeries provides significant insights into its composition, distribution, and potential clinical implications. This study’s findings reveal that PEX material, characterized by its fibrillar and amorphous components, is consistently present across multiple ocular structures, including the anterior lens capsule, eyelid, and conjunctiva. The presence of this material in diverse ocular tissues underscores its pervasive nature and suggests a systemic process rather than a localized phenomenon [19].

**Figure 4:**
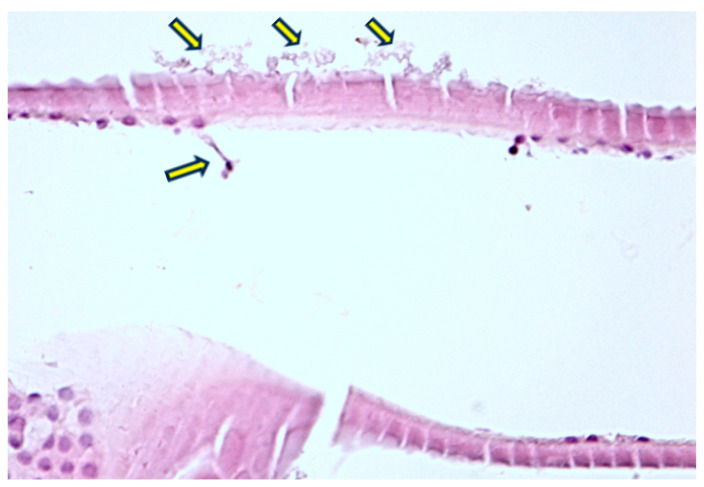
Section through the lens capsule. Note that the stained pseudoexfoliated material (arrows) on the lens confirms the classic “iron filings” appearance with amorphous, eosinophilic deposits. Classic Hematoxylin-Eosin staining, ×400.

The anterior lens capsule exhibited dense accumulations of pseudoexfoliative material, particularly at the pupillary margin, confirming the characteristic “iron fillings” appearance observed clinically (Figure 4). Under higher magnification, the PEX material exhibits a distinctive layered structure, often described as concentric lamellae. Histological staining with hematoxylin-eosin (HE) highlighted the material’s fibrillar texture and glycoprotein content, indicating its complex biochemical composition (Figure 2). The fibrils are composed of microfibrillar proteins and glycoproteins, forming complex aggregates that are resistant to degradation. The PEX material is often interspersed with components of the basement membrane and other extracellular matrix elements, which may contribute to its adhesive properties and its persistence on the lens capsule over time. These findings corroborate previous reports and further support the hypothesis that PEX material originates from abnormal extracellular matrix metabolism within ocular tissues.

**Figure 5:**
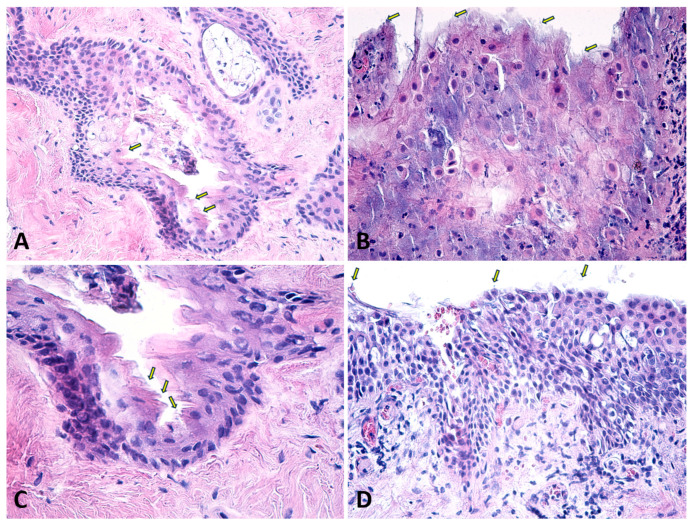
(**A**–**D**) Sections through the epithelium of the lower eyelid. The yellow arrows highlight the presence of pseudoexfoliative material, such as “iron filings”, on the edge of the magnet. When stained with H&E, the PEX material typically appears as amorphous, eosinophilic deposits. Under higher magnification, these deposits exhibit a fibrillar structure, often appearing as irregular, granular, or filamentous aggregates. Classic Hematoxylin-Eosin staining, ×100, ×200.

**Figure 6:**
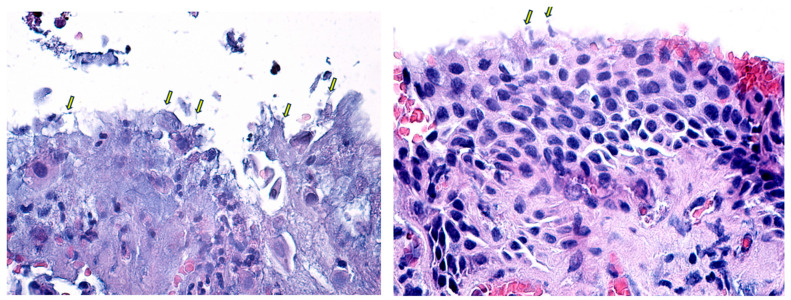
Sections through the epithelium of the lower eyelid. The yellow arrows highlight the presence of psuedoexfoliative material, such as “iron filings”, on the edge of the magnet. When stained with H&E, the PEX material typically appears as amorphous, eosinophilic deposits. Under higher magnification, these deposits exhibit a fibrillar structure, often appearing as irregular, granular, or filamentous aggregates. Classic Hematoxylin-Eosin staining, ×100, ×200.

In the eyelid fragments, pseudoexfoliative material was identified as fine, fibrillar deposits within the extracellular matrix of the eyelid tissues and associated with disrupted basement membranes (Figure 5 and Figure 6). These deposits can be observed in various layers of the eyelid, including the conjunctival epithelium, the tarsal plate, and around blood vessels within the dermis. This distribution pattern suggests that PEX material may contribute to structural changes in the eyelid, potentially influencing the pathogenesis of related ocular surface disorders.

**Figure 7:**
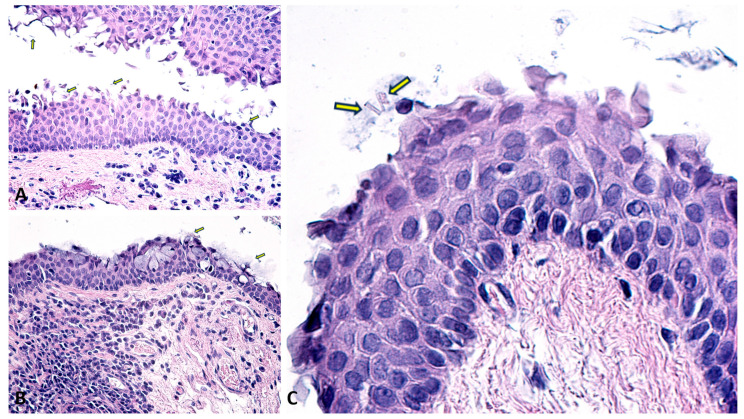
(**A**–**C**) Sections through the conjunctiva. (**A**–**C**) yellow arrows highlight the presence of exfoliative material, such as “iron filings”, on the edge of the magnet. (**C**) PEX structures are seen at the epithelial surface. Classic Hematoxylin-Eosin staining, ×100, ×400.

The conjunctival specimens also showed notable accumulations of PEX material, typically in the stromal layers (Figure 7). The material appears as granular deposits, similar to those observed in other ocular tissues, and may be more diffuse or concentrated in certain areas, depending on the stage and severity of the disease. The fibrils are typically arranged in a random, non-organized pattern, embedded within an amorphous matrix.

## 4. Discussion

The consistent detection of pseudoexfoliative material across various ocular tissues, combined with its characteristic staining patterns, supports the notion that PEX is a multifactorial disorder involving systemic processes such as oxidative stress, inflammation, and impaired extracellular matrix remodeling [15,28,40].

The normal anterior capsule is a membrane with consistent thickness and structure, showing intense staining with PAS, indicating a high concentration of glycosaminoglycans and proteoglycans [41]. The deposition of pseudoexfoliation (PEX) material on the anterior lens capsule is a hallmark of PEX syndrome and is of significant clinical and pathological interest. The anterior lens capsule is one of the primary sites where PEX material accumulates, often presenting as a distinctive “target” or “bull’s-eye” pattern that can be observed clinically and histologically (Figure 1 and Figure 4).

The presence of PEX material on the anterior lens capsule has significant clinical implications, particularly in the context of cataract surgery [42,43]. The firm adhesion of PEX material to the lens capsule can make capsulorhexis more challenging, increasing the risk of complications such as capsule tears or incomplete removal of the lens cortex [44]. Furthermore, PEX deposition on the lens capsule may contribute to the development of cataracts by interfering with normal lens metabolism and inducing oxidative stress within the lens fibers [45]. Finally, the most common surgical complication is caused by zonular instability, which leads to lens subluxation [10,46,47,48].

PEX material in the eyelid is typically found in the dermis and subdermal layers, often associated with elastic fibers and collagen bundles. This material can appear as small, fibrillar deposits, which are morphologically similar to those observed in the anterior segment of the eye. Identifying PXM in the eyelid may contribute to localized tissue changes, such as fibrosis or elastosis, which could affect eyelid function and ocular surface health [49,50,51]. These changes might manifest clinically as eyelid malpositions (e.g., entropion or ectropion) or contribute to meibomian gland dysfunction, leading to ocular surface discomfort and inflammation. All eyelid specimens in our study were collected from patients who requested surgical correction of ectropion or entropion.

The deposit of fibrillar material in the conjunctiva, though less commonly emphasized compared to other structures such as the lens capsule or trabecular meshwork, is a significant finding that sheds light on the systemic nature of the syndrome. The conjunctival PXM deposits may contribute to or be a result of local inflammatory responses, as the conjunctiva is a tissue exposed to environmental factors that could exacerbate or trigger the deposition process [28,52]. Chronic inflammation and oxidative stress are potential mechanisms that could lead to or result from the accumulation of PXM in the conjunctiva [20]. Other conjunctival alterations such as limbic melanotic pigmentation associated with sectorial loss of pupillary ruff were also found to be statistically associated with PEX [53]. A different study comparing conjunctival specimens from PEX eyes and fellow non-PEX eyes found fibrillar material even in the non-PEX eyes, confirming that conjunctival deposits represent a pre-clinical stage [54]. Similarly, Schirmer’s test was statistically lower in PEX patients [51,53]. While conjunctival involvement may not directly lead to the more severe complications seen in the lens or trabecular meshwork, it could still influence ocular surface stability, contribute to dry eye symptoms, or complicate ocular surgeries [49,50,51,55].

It is also important to emphasize the limitations of this study. The absence of a control group without PEX constrains the study’s ability to definitively attribute histopathological findings solely to PEX. Other factors, such as age-related changes, comorbidities, or environmental influences, might have also contributed to the tissue alterations seen in the specimens. This lack of a comparative baseline makes it challenging to isolate PEX as the sole causative factor in the observed pathological changes.

Additionally, there was a significant disparity in the number of specimens collected from different tissue types, which introduces an imbalance that could reduce the robustness of our comparative analyses. Certain tissues were underrepresented in the sample set, limiting the generalizability of findings across various ocular tissues. Since the specimens were obtained during specific ocular surgeries, the surgical procedure itself may have introduced alterations to the tissue, potentially confounding the histopathological outcomes and skewing our interpretation of the results.

Another limitation involves the quality of the specimens. Not all samples met the required criteria for thorough examination. This selection bias might have inadvertently affected the overall findings, as certain subtle or less pronounced histopathological changes could have been missed due to suboptimal tissue quality.

Finally, the small sample size further limits the statistical power of this study. The reduced number of specimens prevented us from identifying statistically significant correlations between the observed tissue changes and factors, such as disease progression, clinical severity, or patient outcomes. As a result, any potential relationships between histopathological findings and the broader clinical context could not be adequately explored, warranting further investigation with larger, more diverse cohorts in future studies.

## 5. Conclusions

This study provides valuable insights into the histopathological characteristics of PXM within various ocular structures, confirming its presence in the anterior lens capsule, eyelid, and conjunctiva. The consistent identification of fibrillar, eosinophilic deposits across these structures highlights the systemic distribution of PEX material, reinforcing the notion that PEX syndrome is not confined to the anterior segment of the eye. The findings suggest that the involvement of the eyelid and conjunctiva, though less frequently reported, plays a crucial role in understanding the broader pathophysiology of PEX. These results underscore the need for further investigation into the systemic implications of PEX and its impact on ocular and extraocular tissues, which could lead to more comprehensive management strategies for patients affected by this condition.

## Figures and Tables

**Table 1:** Pre-operative biometry data of the study population.

Biometry Data	Mean	SD	95% CI
Corneal curvature (D)	44.21	±0.308	43.59–44.83
ACA (grade)	28.80	±1.07	26.65–31.00
ACD (mm)	3.017	±0.07	2.86–3.17
CCT (μm)	541	±4.5	532.23–550.41
Endothelial cell count	2361	±52.73	2255.04–2467.96
% Hexagonal cells	63.76%	± 1.08	61.59–65.93

ACD = anterior chamber depth; ACA = anterior chamber angle; CCT = central corneal thickness; SD = standard deviation; CI = confidence interval.

**Table 2:** Pre-operative biomicroscopic findings in patients with pseudoexfoliation syndrome.

	Characteristic	Percentage
Cornea	Normal	83.05%
	Endothelial fibrillar material	3.75%
	Endothelial decompensation	1.6%
	Endothelial pigment	10%
	Corneal dystrophy	1.6%
Iris	Normal	67.6%
	Pupillary ruff loss	20%
	Radial atrophy	10.8%
	Pigment dispersion in AC	1.6%
	Iridodonesis	0%
Lens	Lens in normal position	100%
TM morphology	Normal	60%
	TM Hyperpigmentation	30%
	Sampaolesi line	10%
Angle	Open	88.75%
	Narrow	11.25%
	Closed	0
Glaucoma	HTIO	7.2%
	OAG	9.6%
	CAG	0%
	Without glaucoma	83.2%

AC = anterior chamber; TM = trabecular meshwork; HTIO = intraocular hypertension; OAG = open-angle glaucoma; CAG = closed-angle glaucoma.

**Table 3:** Best-corrected visual acuity (logMar) of the study population at presentation and post-operative follow-ups.

	Mean	SD	95% CI
BCVA baseline	1.315	±0.747	1.108–1.522
BCVA 7 days	0.164	±0.156	0.121–0.207
BCVA 1 month	0.08	±0.088	0.055–0.104
BCVA 3 months	0.07	±0.083	0.047–0.093

BCVA = best-corrected visual acuity; SD = standard deviation; CI = confidence interval.

**Table 4:** Intraocular pressure of the study population at presentation and post-operative follow-ups.

	Mean	SD	95% CI
IOP baseline	16.42	±4.20	15.25–17.58
IOP 7 days	14.80	±3.42	13.85–15.75
IOP 1 month	14.20	±2.56	13.49–14.91
IOP 3 months	14.08	±2.63	13.35–14.81

IOP = intraocular pressure; SD = standard deviation; CI = confidence interval.

## Data Availability

The datasets used and/or analyzed during the current study are available from the corresponding author upon reasonable request.
